# Sebaceous Carcinoma: A Retrospective Multicenter Analysis of 213 Cases

**DOI:** 10.3390/cancers18081245

**Published:** 2026-04-14

**Authors:** Sebastian A. Wohlfeil, Jochen S. Utikal, Christiane Bauer-Auch, Irina Surovtsova, Tilo Vogel, Anna-Lena Koy, Philipp Morakis

**Affiliations:** 1 Skin Cancer Unit, German Cancer Research Center (DKFZ), 69120 Heidelberg, Germany; 2 Department of Dermatology, Venereology and Allergology, University Medical Center Mannheim, Ruprecht-Karl University of Heidelberg, 68167 Mannheim, Germany; 3 DKFZ Hector Cancer Institute, University Medical Center Mannheim, 68167 Mannheim, Germany; 4 Quality Conferences Office at the Clinical State Registry Baden-Württemberg GmbH, Baden-Württemberg Cancer Registry, 70191 Stuttgart, Germany; 5 Clinical State Registry Baden-Württemberg GmbH, Baden-Württemberg Cancer Registry, 70191 Stuttgart, Germany

**Keywords:** sebaceous carcinoma, adnexal tumor, skin cancer, cutaneous tumor, retrospective study, real-world data

## Abstract

**Background:** Sebaceous carcinoma (SC) is a rare malignant cutaneous malignancy. **Methods:** A multicenter retrospective study of 213 German patients with SC diagnosed between 2008 and 2024 was conducted. Data were extracted from the Baden-Württemberg Cancer Registry. Cases were separated into ocular and extraocular SC. Their demographic, clinical, and treatment-related characteristics were compared and influences on overall survival (OS) analyzed. **Results:** Most patients were elderly (median age: 79 years), with a male-to-female ratio of 2:1. Extraocular SC was more common in men, while ocular SC was more frequent in women. Most tumors were diagnosed at stage I, and microscopically controlled excision was the primary treatment modality (81.4%). Sentinel lymph node biopsy (2.3%), lymph node dissection (1.9%) and systemic therapy (1.4%) were only documented in a minority of cases. Survival analysis (median follow-up 3.2 years) revealed a median OS of 61.4 months in the entire cohort. No significant survival difference was observed between ocular and extraocular SC (64.8 vs. 53.7 months; *p* = 0.490), and multivariable analysis confirmed no prognostic impact of tumor localization (HR 1.4, 95% CI 0.85–2.4). Age was the only independent predictor of outcome, with strongly increased risk in patients aged 70–79 years (HR 4.4, 95% CI 1.01–19.2) and ≥80 years (HR 16.1, 95% CI 3.91–66.1). Prior malignancies, including MTS-like tumors and hematological neoplasms, were not independently associated with overall survival. **Conclusions:** In this multicenter cohort, sebaceous carcinoma showed no survival difference between ocular and extraocular disease, with age emerging as the main independent prognostic factor. Prior malignancies and tumor characteristics, including histologic grade, were not independently associated with outcome. Microscopically controlled excision appears to be an effective treatment option.

## Simple Summary

Sebaceous carcinoma is a rare cutaneous malignancy originating from the adnexal glands of the skin. This retrospective multicenter study analyzed a German cohort of patients with sebaceous carcinoma. The aim was to determine whether findings from patient cohorts in the USA and the UK also apply to our population. The analysis showed that sebaceous carcinoma predominantly occurred in elderly female patients (median age: 79 years) and did not reveal any gender-related differences in overall survival. Despite its reputation as an aggressive adnexal tumor, lymph node excisions or systemic therapy were only performed in a minority of cases. These findings are relevant for the routine management and follow-up of patients with sebaceous carcinoma in Germany and across Europe.

## 1. Introduction

Sebaceous carcinoma (SC) is a rare malignant cutaneous malignancy [1]. Among adnexal tumors at the head and neck it is the most common. In addition, SC is one of the most frequent tumors of the eyelid. SC of the skin is classified into a periocular subtype, accounting for around 75% of the cases, and an extraocular subtype [2]. Clinically, SC often presents as a fast growing, yellow-colored, ulcerated nodule [3].

The population at risk are patients older than 60 years, but also a few pediatric cases with SC were reported [4]. Epidemiologic analyses show a strong increase in recent years, mostly in male patients with fair skin [5]. The incidence ranges from 0.1 per 100.000 person-years in England to 0.23 in the US [5,6]. There is still a controversy regarding overall gender-specific incidences [4,5]. Nevertheless, women tend to be diagnosed more often with periocular SC [5].

The exact pathophysiology remains elusive. Both UV irradiation and radiotherapy may be causative risk factors [7]. In addition, patients with immunosuppression, caused by hematological malignancies, infection with human immunodeficiency virus (HIV) or immunosuppressants, were prone to develop SC [7]. Furthermore, viral oncogenesis, such as human papilloma virus, Merkel cell polyoma virus and Epstein–Barr virus, were discussed as explanatory factors of SC [8].

Molecular profiling defines three subtypes: UV-mutated, pauci-mutated and microsatellite instable (MSI) SC [9]. Periocular SC are often pauci-mutated, while extraocular SC show UV signatures or MSI. Potential drivers of SC include mutations along the phosphoinositide 3-kinase (PI3K) pathway [10]. Periocular SC specifically often shows mutations of the TP53 and RB1 genes. In contrast, extraocular SC reveal hereditary mutations, MSI or mismatch repair (MMR) genes.

Interestingly, SC also occurs as part of the Muir–Torre syndrome (MTS) which is a phenotypic variant of the Lynch syndrome [11]. It results from autosomal dominant inherited mutations in MMR genes, such as MSH2. Patients with this condition are prone to develop sebaceous neoplasms, including SC, or visceral malignancies like colorectal carcinoma. Typically, periocular SC is not associated with MTS. The Mayo MTS risk scoring may help to screen patients with extraocular SC for MTS [12]. It includes the age of the patient, number of sebaceous tumors and malignancies associated with Lynch syndrome in their own and family history. Genetic testing should be performed in patients with a score ≥ 2.

The primary treatment of SC is surgery, with micrographic surgery as the gold standard [1,13]. Numerous studies showed that micrographic surgery is superior to local wide excision which was often performed in the past [1,14,15,16]. In addition, this is an advantage for aesthetic areas such as the eyelid [17]. For periocular SC stage T2c and higher, but not for extraocular SC, sonography of the lymph nodes (LN) and sentinel lymph node (SLN) biopsy are recommended [1,13,18]. If nodal involvement was detected, a LN dissection should be discussed with the patient. SC are classified according to the UICC classification for skin cancer and its respective localization.

Radiotherapy is considered an option for patients who decline surgical treatment [19]. Data on adjuvant radiotherapy is limited but may be performed in SC with certain risk factors such as incomplete resection, recurrent lesions or perineural invasion [19,20]. For systemic treatment of SC, no randomized controlled clinical trials have been performed, as only few cases with metastasized SC have been reported. Systemic treatment comprised conventional chemotherapy or immune checkpoint inhibition [21,22]. Currently, enfortumab vedotin, an antibody-drug conjugate, is being discussed as a novel systemic therapy of SC due to its expression of Nectin-4 [23]. Neoadjuvant application of a platinum-based chemotherapy proved effective for periocular SC in a case series [24].

Recent analyses of the American National Cancer Database report an increased mortality in extraocular compared to periocular SC [5], while old analyses did not find any differences [4]. Therefore, our multicenter retrospective study on SC compared real-world data from German cancer centers to add evidence on the presentation and clinical course of SC in Europe.

## 2. Materials and Methods

### 2.1. Data Collection and Study Population

A retrospective multicenter analysis was performed on a data set obtained from the clinical Cancer Registry Database (Klinisches Landeskrebsregister, KLR GmbH) of the German Federal State Baden Württemberg (BW). Cases with diagnosis of SC between 2008 and 2024 were extracted from the registry. Data cutoff was on the 1st of December 2024. All cases with histologically confirmed SC (ICD-O-3 code 8410/3) were extracted. Tumor localization was defined according to ICD-10 codes and categorized into ocular and extraocular sites. Ocular tumors were restricted to the eyelid (C44.1). Extraocular tumors included lesions of the head and neck region (C44.0, C44.2, C44.3 and C44.4) as well as tumors of non-head and neck sites, including trunk, extremities, and genital regions (C44.5*, C44.6, C44.7, C44.8, C44.9, C51*, C63.2).

Nine cases with a multiple synchronous SC were excluded from the analysis.

### 2.2. Statistical Analyses

Baseline characteristics were compared using Fisher’s exact test or the chi-square test, as appropriate. Only patients residing in Baden-Württemberg were included in the survival analyses. Overall survival (OS) was defined as time from diagnosis to death from any cause. Survival outcomes were analyzed using Kaplan–Meier estimates with the log-rank test, while multivariable Cox proportional hazards models were applied to assess independent prognostic factors. Results are presented as median OS rates and hazard ratios (HRs) with 95% confidence intervals (CIs). Statistical significance was set at *p* < 0.05.

All statistical analyses were performed with R (version 4.2.1) and the tidyverse, survival or survminer packages.

## 3. Results

**Figure 1:**
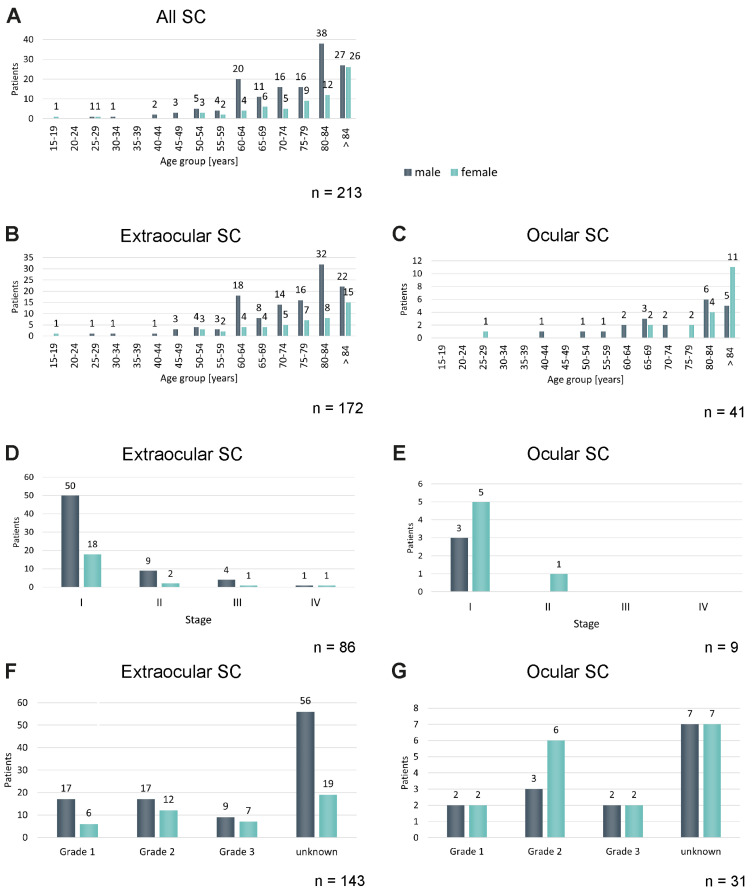
Characteristics of SC. (**A**) The age of the patients at diagnosis of SC has been compared. The number of patients in the corresponding age group is displayed. Male patients are presented in dark turquoise, and female patients in bright turquoise. *p* = 0.139 (Chi^2^-Test), n = 213. (**B**) The age of patients with extraocular SC has been compared. The number of patients in the corresponding age group is displayed. Male patients are presented in dark turquoise, and female patients in bright turquoise. *p* = 0.498 (Chi^2^-Test), n = 172. (**C**) The age of patients with ocular SC has been compared. The number of patients in the corresponding age group is displayed. Male patients are presented in dark turquoise, and female patients in bright turquoise. *p* = 0.170 (Chi^2^-Test), n = 41. (**D**) The tumor stage of patients with extraocular SC has been compared. The number of patients in the corresponding age group is displayed. Male patients are presented in dark turquoise, and female patients in bright turquoise. *p* = 0.402 (Chi^2^-Test), n = 86. (**E**) The tumor stage of patients with ocular SC has been compared. The number of patients in the corresponding age group is displayed. Male patients are presented in dark turquoise, and female patients in bright turquoise. *p* = 0.294 (Chi^2^-Test), n = 9. (**F**) The histological grade of extraocular SC has been compared. The number of patients in the corresponding age group is displayed. Male patients are presented in dark turquoise, and female patients in bright turquoise. *p* = 0.321 (Chi^2^-Test), n = 143. (**G**) The histological grade of ocular SC has been compared. The number of patients in the corresponding age group is displayed. Male patients are presented in dark turquoise, and female patients in bright turquoise. *p* = 0.290 (Chi^2^-Test), n = 31.

A total of 213 patients were included, comprising 172 with extraocular and 41 with ocular tumors (Figure 1, Table 1).

The extraocular tumors were most commonly located in C44.3 (81; 38.0%), followed by C44.4 (35; 16.4%), C44.5 (21; 9.9%), C44.2 (13; 6.1%), and C44.6 (10; 4.7%). Less frequent sites included C44.9 (5) and C44.7 (3), while all other locations (C44.0, C44.59, C51.9, C63.2) were rare (n = 1 each) (for a detailed mapping of ICD-10 codes to anatomical locations please refer to Supplementary Table S1).

The mean age of the overall cohort was 75.1 years (extraocular: 74.5; ocular: 77.5; *p* = 0.213), with a median age of 79 years (extraocular: 77.5; ocular: 83; *p* = 0.054). Age distribution did not differ significantly between groups (*p* = 0.087), although patients older than 80 years were more frequent in the ocular group (63.4% vs. 44.8%), (Figure 1A–C, Table 1). Male patients predominated overall (67.6%), with a significantly higher proportion in the extraocular group compared to the ocular group (71.5% vs. 51.2%; *p* = 0.021).

Tumor grading, T-, N-, and M-stage, as well as UICC stage, were available for a subset of patients and were analyzed descriptively. Based on available UICC staging, the majority of patients presented with stage I disease (80.0%), with no significant differences between ocular and extraocular groups (*p* = 0.838), (Figure 1D,E). Only five cases of extraocular SC were in stage III (Supplementary Table S2), and two were in stage IV (Supplementary Table S3).

Among cases with available data, tumor grading was comparable between groups (*p* = 0.638), with grade 2 being the most frequent (44.2%). Both extraocular and ocular SC showed no obvious differences regarding grading of SC in male and female patients (Figure 1F,G). The T stage tended to be more advanced in ocular tumors; this difference did not reach statistical significance (*p* = 0.055). Mean tumor thickness was 3.92 mm overall and did not differ significantly between extraocular and ocular tumors (4.04 vs. 2.98 mm; *p* = 0.554).

Regarding nodal or distant metastasis only few cases were reported (Table 1). Nodal disease occurred more frequently in ocular SC than in extraocular SC (*p* = 0.03), while there was no difference regarding distant metastasis (Table 1). However, this must be interpreted with caution, given the small number of ocular SC cases.

**Figure 2:**
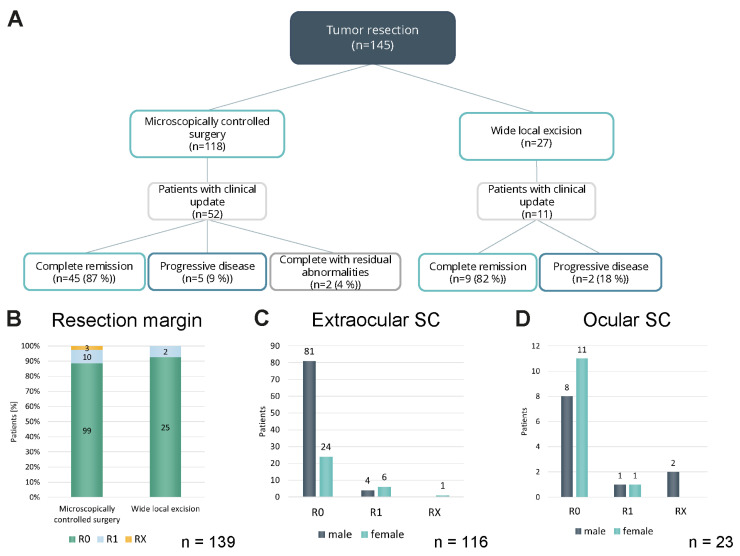
Surgical treatment of SC at initial presentation. (**A**) Flow chart showing the surgical treatment of SC patients at first presentation and clinical update data, if available. (**B**) Bar chart comparing the resection margin of SCs after microscopically controlled surgery vs. wide local excision. (**C**) Bar chart displaying the resection margins of extraocular SCs in male and female patients after surgical treatment. (**D**) Bar chart showing the resection margins of ocular SCs in male and female patients after surgical treatment.

Treatment of SCs was mainly surgical (Figure 2A). In 81.4% (118 cases), a microscopically controlled excision was performed. When comparing margin-free (R0) resections, results were similar to wide local excisions (Figure 2B). However, the exact safety margins of the wide local excisions had not been documented in the registry data. Regarding extraocular SC, the percentage of margin-free resected SC (R0) was slightly higher in male (95.3%) as compared to female (77.4%) patients (Figure 2C). In ocular SC a lower percentage of complete tumor resections (R0) was found in male patients (72.7%) in relation to female patients (91.7%) (Figure 2D). Among 63 patients with available postoperative outcome data, those primarily treated with microscopically controlled surgery showed a lower rate of progressive disease (9%; five cases) compared to patients with wide local excision (18%; two cases) (Figure 2A). However, the number of patients treated with wide local excision was low (27 cases in total).

In only 2.3% of all cases, specifically five cases with extraocular SC, a SLN biopsy was performed (Supplementary Table S4). A LN dissection was only documented in 1.9% of our cohort (four patients), with no difference between extraocular and ocular SC patients (Supplementary Table S4). Radiotherapy was performed in two cases as definitive therapy for extraocular stage III and IV SC, and four SC cases received an adjuvant radiotherapy (Supplementary Table S5). Systemic therapy with platinum-based chemotherapy or taxanes, often in combination with radiotherapy, was only performed in three patients, two patients with locoregional disease and one case with lung metastasis (Supplementary Figure S1A).

Overall survival analysis was further conducted to explore factors associated with prognosis in patients with SC. This analysis was restricted to patients residing in Baden-Württemberg (n = 186), with a median observation time of 3.2 years. The median OS in the entire cohort was 61.4 months (95% CI 46.8–95.8).

**Figure 3:**
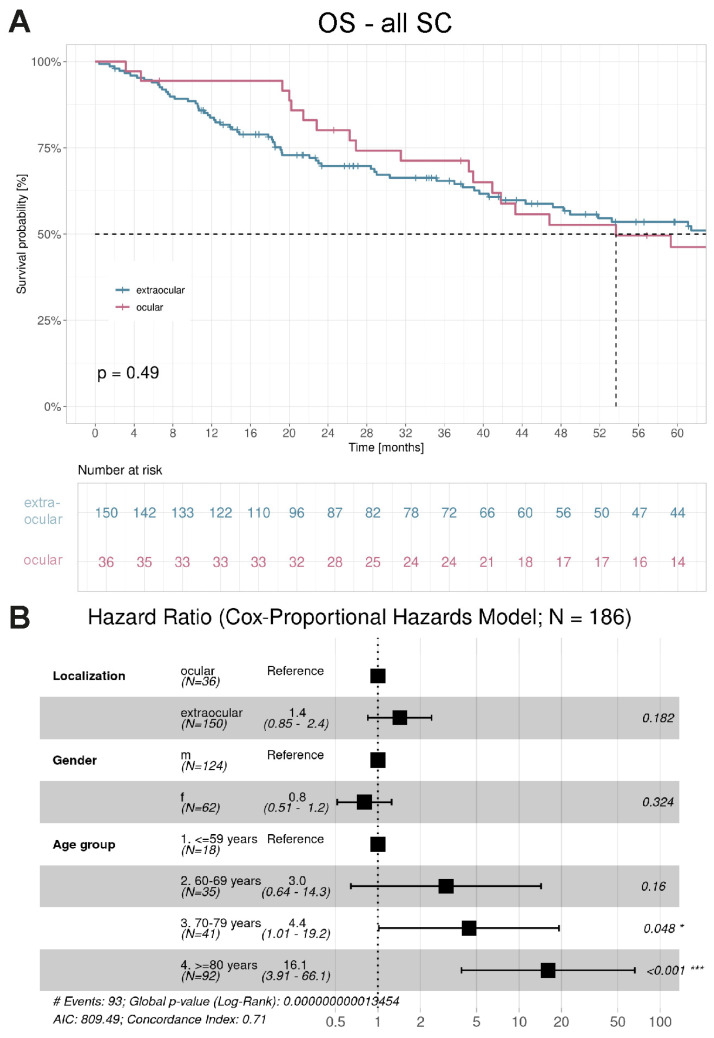
The 5-year OS of patients with SC. (**A**) The 5-year OS of patients with extraocular and ocular SC was compared. Follow-up started from the date of diagnosis of SC. Kaplan–Meier curves are shown; extraocular SC cases are presented in blue and ocular SC cases in red. The curves were compared by log-rank tests (*p* = 0.490). The dashed line displays the median OS time. (**B**) Multivariable Cox proportional hazards analysis of OS. Tumor localization was not associated with survival (extraocular vs. ocular: HR 1.4, 95% CI 0.85–2.4; *p* = 0.182). Increasing age was the strongest independent prognostic factor (70–79 years: HR 4.4, 95% CI 1.01–19.2; ≥80 years: HR 16.1, 95% CI 3.91–66.1), while sex had no significant impact. * *p* < 0.05; *** *p* < 0.001.

We first assessed whether tumor localization at initial presentation influenced the OS. Patients with extraocular SC had a median OS of 64.8 months compared to 53.7 months in those with ocular SC, with no significant difference observed between groups (*p* = 0.490) (Figure 3A). Multivariable Cox regression analysis confirmed the findings of the univariate analysis, showing no significant association between tumor localization and overall survival (extraocular vs. ocular: HR 1.4, 95% CI 0.85–2.4; Figure 3B). Age remained a strong independent prognostic factor: compared to patients <60 years, those aged 70–79 years had an HR of 4.4 (95% CI 1.01–19.2), and those aged ≥80 years an HR of 16.1 (95% CI 3.91–66.1).

For extraocular and ocular SC there were also no gender-specific differences among the patients (Supplementary Figure S2A,B). Second, the histologic grading was investigated. No differences were observed among patients with SC from grade 1 to 3 (Supplementary Figure S3A).

**Figure 4:**
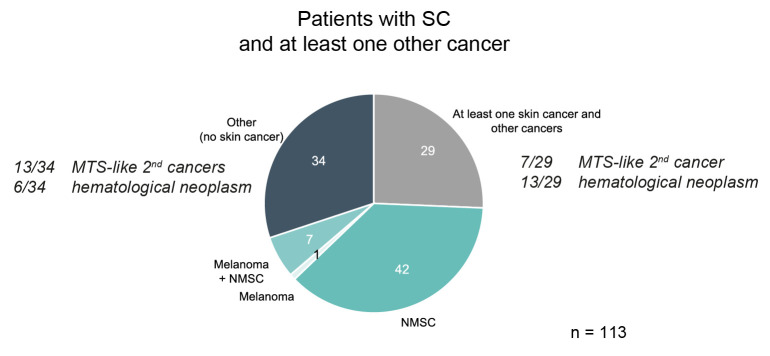
Prior cancer history and influence on disease course of SC. A pie chart showing SC patients who had a history of cancer(s) prior to diagnosis of SC. These were separated into skin cancers, such as melanoma and non-melanoma skin cancer (NMSC). In addition, SC patients diagnosed with other malignancies were grouped into “other (without skin cancer)”, and a group who suffered both from skin cancers and other malignancies. These groups were further separated into cancer entities which might be associated with MTS, referred to as “MTS-like” cancers, and hematological neoplasia.

As SC might also occur as part of MTS [11], we were interested in secondary malignancies. The Mayo MTS score is a helpful tool to identify patients at risk [12]. Unfortunately, due to the retrospective analysis of our study and the nature of the registry data, this could not be applied to our cohort. Therefore, we selected patients who suffered from malignancies before diagnosis of SC. Patients with multiple SCs were excluded from the whole study (Supplementary Table S6). In total, 113 cases had a history of a malignancy before the diagnosis of SC (Figure 4). Commonly, this was non-melanoma skin cancer (NMSC) (37.2%; 42 cases).

A total of 34 patients (30.0%) had at least one additional non-cutaneous malignancy (Supplementary Table S7) including 13 cases (38.2%) with cancers like bladder cancer or rectal cancer that might be associated with MTS (MTS-like) and six cases (17.6%) with hematological neoplasia, such as lymphoma or leukemia, were identified.

In addition, 29 patients (25.7%) had a broader history of malignancies including skin cancers (C44) (Supplementary Table S8). Within this subgroup, seven (24.1%) had MTS-like cancers and 13 patients (44.8%) had hematological malignancies.

**Figure 5:**
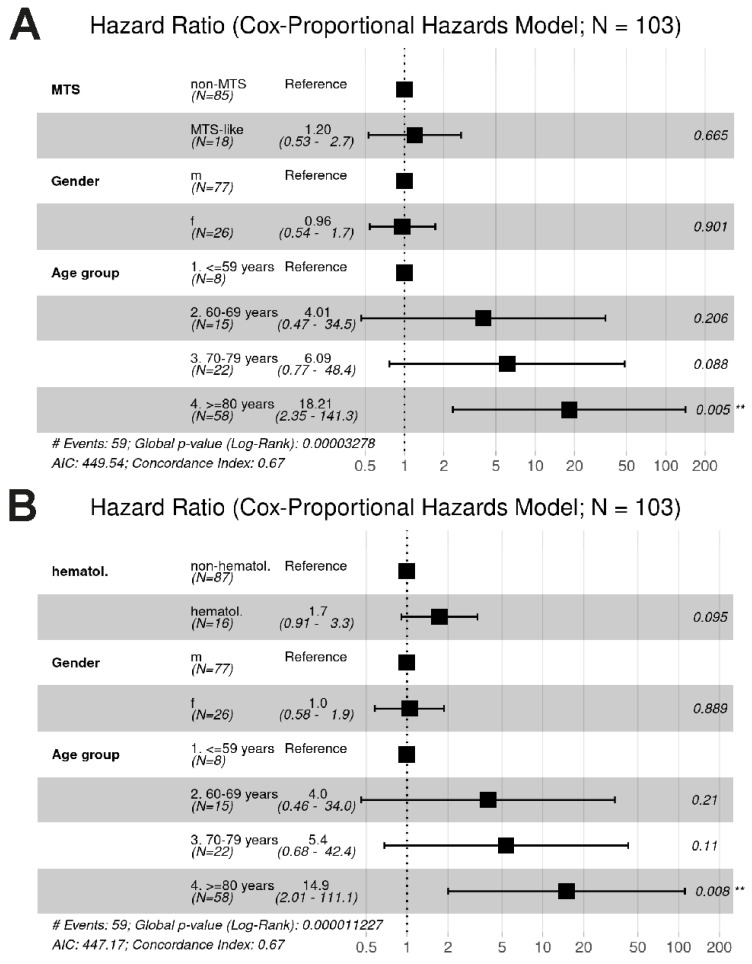
Multivariable Cox proportional hazards analyses of overall survival. (**A**) Association of MTS-like malignancies with overall survival. MTS-like cancers were not significantly associated with survival compared to non-MTS malignancies. (**B**) Association of hematological malignancies with overall survival. A trend toward worse survival was observed for patients with hematological malignancies, although this did not reach statistical significance. In both models, increasing age was the strongest independent predictor of poorer survival, while sex showed no significant effect. The dashed horizontal lines at hazard ratio 1.0 correspond to no difference in risk between the groups. ** *p* < 0.01.

We further assessed the impact of prior malignancies on overall survival. In univariate analysis, patients with MTS-like cancers showed a trend toward improved survival compared to those with other malignancies (*p* = 0.073; Supplementary Table S9 and Supplementary Figure S4A), although this did not reach statistical significance and was not confirmed in multivariable Cox regression (HR 1.2, 95% CI 0.53–2.7; Figure 5A).

Hematological neoplasms were associated with significantly worse OS in univariate analysis compared with patients with other prior malignancies (*p* = 0.0056, Supplementary Table S10 and Supplementary Figure S4B), but this association was not retained in multivariable analysis (HR 1.7, 95% CI 0.91–3.3; Figure 5B).

## 4. Discussion

Since SC is a rare adnexal tumor [1], data from large patient cohorts remain limited. Therefore, our multicenter retrospective study aimed at analyzing the epidemiology, treatment and outcome of 213 patients with either extraocular or ocular SC in the German federal state of Baden-Württemberg, which has a population of over 11 million (as of 2019).

SC predominantly affected the elderly with a median age of 79 years—higher than that reported in previous reports [4,25,26]. In addition, our data revealed a male-to-female ratio of 2:1 among all SC patients. Published studies confirm this predominance in men [5,25,26]. The incidence in men seems to be rising in comparison to earlier data from the Surveillance, Epidemiology, and End Results (SEER) program [4]. However, this could be biased by enhanced histopathological diagnostics and disease awareness. With respect to the localization, extraocular SC was more common in elderly men, while ocular SC was overrepresented in female patients older than 65 years. Epidemiologic data from the USA, England, Norway or Taiwan are in line with our findings [6].

As recommended by guidelines [1,13], primary treatment of SC was surgical, with microscopically controlled surgery used in 81.4%. Follow-up data suggest a benefit for microscopically controlled surgery (9% recurrence) as compared to wide local excision (18.6% recurrence). Yadlapati et al. confirmed a superiority of Mohs micrographic surgery to wide local excision regarding local and distant recurrence in a systematic review [15]. In ocular SC, nodal disease was significantly more frequent than in extraocular SC which is in line with the literature [27]. Extended procedures such as SLN biopsy were only performed in 2.3% and LN dissection in 1.9% of all cases. Furthermore, only one case with lung metastatic SC was documented in our cohort and two cases with extended locoregional metastasis which were treated by systemic therapy with platinum-based chemotherapy or taxanes. One patient with stage IV SC (pT4b) received radiotherapy. It appears that SC is indeed a potentially aggressive adnexal tumor [13], but the metastasis rate would be low if it were adequately detected and treated. This notion is also supported by Chinese and Dutch cohorts that did not even detect any metastases in SC smaller than 10 mm [26,28].

The most important prognostic factor remains age. Especially, SC patients older than 70 years showed significantly decreased OS [29]. Notably, a recent study demonstrates that the increased mortality among SC patients is especially driven by cardiovascular, cerebrovascular, metabolic, kidney and lung diseases [30]. This is conceivable given the advanced age of the patients of SC study cohorts.

Furthermore, solid organ transplantation is associated with a 25-fold increased risk to develop SC [7]. It is hypothesized that viral oncogenesis, immunosuppressive agents, medication inducing photosensitization or underlying medical conditions increase the risk of patients with solid organ transplantation to develop SC [31]. In this context, our findings suggest that immune dysregulation may also influence outcomes beyond the transplant setting. Patients with a history of hematological malignancies showed worse overall survival compared to those with other prior cancers, despite similar age distributions, possibly reflecting impaired tumor surveillance due to persistent immune alterations. However, this association was observed only in univariate analysis and was not confirmed in the multivariable model, indicating potential confounding or limited statistical power. These findings should therefore be interpreted with caution.

A comparison of the localization of SC in our study showed that the OS was similar between ocular and extraocular SC patients, in contrast to the study by Tripathi and colleagues who detected a 1.4-fold increased mortality of extraocular SC patients in relation to ocular SC patients [5]. In addition, no gender-specific differences in overall survival were detected, which is also seen in cohorts from England [25] and in a SEER data set with ocular SC from the USA [29]. Last, no influence of the tumor grading on the OS was observed, as in a cohort of ocular SC [29]. Lately, it was debated whether MTS affects the OS of patients with SC [32,33]. Therefore, we analyzed the OS of SC patients with history of malignancies associated with MTS, such as gastrointestinal or urological cancers. This analysis was limited as neither information on family history nor genetic analyses were available. In our study we observed a tendency toward an improved OS in patients with SC and history of MTS-like cancers as compared to patients with SC and other malignancies. However, this likely reflects confounding by age, as these patients were significantly younger than the comparison group. Importantly, no association was observed in the multivariable model, indicating that MTS-like malignancies were not independently associated with survival in our cohort. Our data is in accordance with a study from Maloney and colleagues [33].

Our multicenter retrospective analysis represents a relatively large cohort of patients with sebaceous carcinoma; however, several limitations should be acknowledged. As is inherent to retrospective registry studies, documentation bias is likely, with initial diagnostic information generally well captured but subsequent clinical details sometimes incomplete. In addition, several relevant variables—including margin status, molecular features, adverse events, and comorbidities—were not systematically recorded. Furthermore, heterogeneity in treatment approaches across participating centers, including aspects of supportive care, may have influenced outcomes and limits direct comparability between groups.

Despite these limitations, the large sample size and population-based design enhance the robustness of our findings and provide valuable insight into real-world clinical outcomes. External validation in independent cohorts is desirable and remains beyond the scope of the present study.

## 5. Conclusions

In this relatively large multicenter cohort, sebaceous carcinoma mainly affected elderly patients and showed no survival difference between ocular and extraocular disease, with age being the only independent prognostic factor. Prior malignancies were common, including MTS-like and hematological tumors, but none showed an independent impact on survival despite divergent univariate trends.

## Figures and Tables

**Table 1:** Baseline characteristics of study population stratified by tumor localization (ocular vs. extraocular).

Variable		Overall	Extraocular	Ocular	*p* Value
n		213	172	41	
Age (mean)		75.1	74.5	77.5	0.213
Age (median…range)		79.0 (19–97)	77.5 (19–97)	83.0 (27–94)	0.054
Age group	<60	23 (10.8%)	19 (11.0%)	4 (9.8%)	0.087
	60–79	87 (40.8%)	76 (44.2%)	11 (26.8%)	
	≥80	103 (48.4%)	77 (44.8%)	26 (63.4%)	
Gender	m	144 (67.6%)	123 (71.5%)	21 (51.2%)	0.021
	f	69 (32.4%)	49 (28.5%)	20 (48.8%)	
Grading	1	28 (32.6%)	24 (34.8%)	4 (23.5%)	0.638
	2	38 (44.2%)	29 (42.0%)	9 (52.9%)	
	3	20 (23.3%)	16 (23.2%)	4 (23.5%)	
Tumor status	T1	104 (77.6%)	91 (82.0%)	13 (56.5%)	0.055
	T2	21 (15.7%)	14 (12.6%)	7 (30.4%)	
	T3	7 (5.2%)	5 (4.5%)	2 (8.7%)	
	T4	2 (1.5%)	1 (0.9%)	1 (4.3%)	
Nodal status	N0	106 (95.5%)	93 (96.9%)	13 (86.7%)	0.030
	N1	4 (3.6%)	3 (3.1%)	1 (6.7%)	
	N2	1 (0.9%)	0 (0%)	1 (6.7%)	
Metastases	M0	104 (99.0%)	93 (98.9%)	11 (100.0%)	1.000
	M1	1 (1.0%)	1 (1.1%)	0 (0%)	
Stage (UICC)	I	76 (80.0%)	68 (79.1%)	8 (88.9%)	0.838
	II	12 (12.6%)	11 (12.8%)	1 (11.1%)	
	III	5 (5.3%)	5 (5.8%)	0 (0.0%)	
	IV	2 (2.1%)	2 (2.3%)	0 (0.0%)	
Resection margin	R0	124 (91.2%)	105 (91.3%)	19 (90.5%)	1.000
(R status)	R1	12 (8.8%)	10 (8.7%)	2 (9.5%)	
Tumor thickness (±SD)		3.92 (3.33)	4.04 (3.35)	2.98 (3.41)	0.554

## Data Availability

All relevant data is contained within the article: The original contributions presented in the study are included in the article/Supplementary Material, further inquiries can be directed to the corresponding authors.

## Supplementary Materials

The following supporting information can be downloaded at: https://www.mdpi.com/article/10.3390/cancers18081245/s1. Figure S1: Flow chart of patients with systemic therapy. Figure S2: Gender-specific OS of extraocular and ocular SC patients. Figure S3: Sebaceous carcinoma. Influence of histologic grade on OS. Figure S4: Prior cancer history and influence on disease course of SC. Table S1: Grouping according to ICD-10 codes. Table S2: Cases with SC in stage III. Table S3: Cases with SC in stage IV. Table S4: Cases with LN excision. Table S5: Cases with radiotherapy. Table S6: Cases with multiple SC. Table S7: Cases with SC and at least another malignancy, apart from skin tumors. Table S8: Cases with SC and at least another malignancy, including skin tumors (C44). Table S9: Baseline characteristics of MTS-like and non-MTS cohorts. Table S10: Baseline characteristics of SC patients with a history of hematological and non-hematological neoplasms.

## Abbreviations

The following abbreviations are used in this manuscript:

BWCR	Baden-Württemberg Cancer Registry
Hematol.	hematological
HIV	human immunodeficiency virus
LN	lymph node
MMR	mismatch repair
MSI	microsatellite instable/instability
MTS	Muir-Torre syndrome
NMSC	non-melanoma skin cancer
PI3K	phosphoinositide 3-kinase
SC	sebaceous carcinoma
SEER	Surveillance, Epidemiology, and End Results
SLN	sentinel lymph node
UICC	Union internationale contre le cancer

## References

[1] Owen J.L., Kibbi N., Worley B., Kelm R.C., Wang J.V., Barker C.A., Behshad R., Bichakjian C.K., Bolotin D., Bordeaux J.S. (2019). Sebaceous carcinoma: Evidence-based clinical practice guidelines. Lancet Oncol..

[2] Elder D.E., Massi D., Scolyer R.A., Willemze R. (2018). WHO Classification of Skin Tumours: WHO Classification of Tumours.

[3] Buitrago W., Joseph A.K. (2008). Sebaceous carcinoma: The great masquerader: Emgerging concepts in diagnosis and treatment. Dermatol. Ther..

[4] Dasgupta T., Wilson L.D., Yu J.B. (2009). A retrospective review of 1349 cases of sebaceous carcinoma. Cancer.

[5] Tripathi R., Chen Z., Li L., Bordeaux J.S. (2016). Incidence and survival of sebaceous carcinoma in the United States. J. Am. Acad. Dermatol..

[6] Wu A., Rajak S.N., Chiang C.J., Lee W.C., Huilgol S.C., Selva D. (2021). Epidemiology of cutaneous sebaceous carcinoma. Australas. J. Dermatol..

[7] Sargen M.R., Cahoon E.K., Lynch C.F., Tucker M.A., Goldstein A.M., Engels E.A. (2020). Sebaceous Carcinoma Incidence and Survival Among Solid Organ Transplant Recipients in the United States, 1987–2017. JAMA Dermatol..

[8] Sargen M.R., Starrett G.J., Engels E.A., Cahoon E.K., Tucker M.A., Goldstein A.M. (2021). Sebaceous Carcinoma Epidemiology and Genetics: Emerging Concepts and Clinical Implications for Screening, Prevention, and Treatment. Clin. Cancer Res..

[9] North J.P., Golovato J., Vaske C.J., Sanborn J.Z., Nguyen A., Wu W., Goode B., Stevers M., McMullen K., Perez White B.E. (2018). Cell of origin and mutation pattern define three clinically distinct classes of sebaceous carcinoma. Nat. Commun..

[10] Tetzlaff M.T., Singh R.R., Seviour E.G., Curry J.L., Hudgens C.W., Bell D., Wimmer D.A., Ning J., Czerniak B.A., Zhang L. (2016). Next-generation sequencing identifies high frequency of mutations in potentially clinically actionable genes in sebaceous carcinoma. J. Pathol..

[11] Schwartz R.A., Torre D.P. (1995). The Muir-Torre syndrome: A 25-year retrospect. J. Am. Acad. Dermatol..

[12] Roberts M.E., Riegert-Johnson D.L., Thomas B.C., Rumilla K.M., Thomas C.S., Heckman M.G., Purcell J.U., Hanson N.B., Leppig K.A., Lim J. (2014). A clinical scoring system to identify patients with sebaceous neoplasms at risk for the Muir-Torre variant of Lynch syndrome. Genet. Med..

[13] Utikal J., Nagel P., Muller V., Becker J.C., Dippel E., Frisman A., Gschnell M., Griewank K., Hadaschik E., Helbig D. (2024). S1-Guideline Sebaceous Carcinoma. J. Dtsch. Dermatol. Ges..

[14] Zhou C., Wu F., Chai P., Shi Y., Ye J., Shi X., Tan J., Ding Y., Luo Y., Esmaeli B. (2019). Mohs micrographic surgery for eyelid sebaceous carcinoma: A multicenter cohort of 360 patients. J. Am. Acad. Dermatol..

[15] Yadlapati S., Rosa-Nieves P.M., Mehta N., Merritt B.G., Carrasquillo O.Y. (2024). Treatment of sebaceous carcinoma with Mohs micrographic surgery versus wide local excision: A systematic review. Int. J. Dermatol..

[16] Joshi T.P., Wu A.X., Kimyai-Asadi A. (2025). Enhanced disease-specific survival following Mohs micrographic surgery compared to wide local excision for sebaceous carcinoma: A national database study. Int. J. Dermatol..

[17] Xu M., Chen Q., Luo Y., Chai P., He X., Huang H., Tan J., Ye J., Zhou C. (2024). Recurrence in Eyelid Sebaceous Carcinoma: A Multicentric Study of 418 Patients. Investig. Ophthalmol. Vis. Sci..

[18] Lu T., Anthony C., Jiang X., Ning J., Goepfert R., Esmaeli B. (2025). Timing of nodal metastasis in patients with eyelid sebaceous carcinoma and implications for surveillance and survival. Br. J. Ophthalmol..

[19] Mohamed N., Mattessich S., Gelblum D.Y., Lee N.Y., Barker C.A. (2025). Treatment of Cutaneous Adnexal Carcinoma With Radiotherapy: A 20-Year, Single-Institution Experience in 49 Patients. Am. J. Clin. Oncol..

[20] Giridhar P., Kashyap L., Mallick S., Dutt Upadhyay A., Rath G.K. (2020). Impact of surgery and adjuvant treatment on the outcome of extraocular sebaceous carcinoma: A systematic review and individual patient’s data analysis of 206 cases. Int. J. Dermatol..

[21] Domingo-Musibay E., Murugan P., Giubellino A., Sharma S., Steinberger D., Yuan J., Hunt M.A., Lou E., Miller J.S. (2018). Near complete response to Pembrolizumab in microsatellite-stable metastatic sebaceous carcinoma. J. Immunother. Cancer.

[22] Orcurto A., Gay B.E., Sozzi W.J., Gilliet M., Leyvraz S. (2014). Long-Term Remission of an Aggressive Sebaceous Carcinoma following Chemotherapy. Case Rep. Dermatol..

[23] Cho W.C., Saade R., Nagarajan P., Aung P.P., Milton D.R., Marques-Piubelli M.L., Hudgens C., Ledesma D., Nelson K., Ivan D. (2024). Nectin-4 expression in a subset of cutaneous adnexal carcinomas: A potential target for therapy with enfortumab vedotin. J. Cutan. Pathol..

[24] Vempuluru V.S., Luthra A., Reddy Palkonda V.A., Kaliki S. (2025). Multimodal treatment with Neoadjuvant chemotherapy for eyelid and periocular sebaceous gland carcinoma: Long-term outcomes. Am. J. Ophthalmol. Case Rep..

[25] Cook S., Pethick J., Kibbi N., Hollestein L., Lavelle K., de Vere Hunt I., Turnbull C., Rous B., Husain A., Burn J. (2023). Sebaceous carcinoma epidemiology, associated malignancies and Lynch/Muir-Torre syndrome screening in England from 2008 to 2018. J. Am. Acad. Dermatol..

[26] In ’t Veld E.H., Keizer R., Post N., Versteeg J., Verdijk R., Naus N., Relyveld G., Crijns M., Smith M., Grünhagen D. (2022). Outcome after treatment for sebaceous carcinoma: A multicenter study. J. Surg. Oncol..

[27] Tryggvason G., Bayon R., Pagedar N.A. (2012). Epidemiology of sebaceous carcinoma of the head and neck: Implications for lymph node management. Head Neck.

[28] Lam S.C., Li E.Y.M., Yuen H.K.L. (2018). 14-year case series of eyelid sebaceous gland carcinoma in Chinese patients and review of management. Br. J. Ophthalmol..

[29] Yasinzai A.Q.K., Goodbee M., Ahweyevu J., Tareen A.S.K., Ullah H., Tareen M.A., Waheed A., Karim A., Arif D., Khan M. (2023). Sebaceous carcinoma of the eyelid: Demographical analysis and role of surgery in the management. Eur. J. Plast. Surg..

[30] Joshi T.P., Ranario J.S. (2025). Overall drivers of mortality in sebaceous carcinoma patients with and without Muir-Torre syndrome: A population-based analysis. J. Am. Acad. Dermatol..

[31] Tripathi R., Nijhawan R.I., Bordeaux J.S. (2023). Sebaceous carcinoma in solid organ transplant recipients: The elegant path from epidemiology to etiology. Cancer Epidemiol..

[32] Tripathi R., Bordeaux J.S. (2019). Impact of Muir-Torre Syndrome on Survival in Patients With Sebaceous Carcinoma: A SEER Population-Based Study. Dermatol. Surg..

[33] Maloney N.J., Zacher N.C., Hirotsu K.E., Rajan N., Aasi S.Z., Kibbi N. (2023). Comparison of clinicopathologic features, survival, and demographics in sebaceous carcinoma patients with and without Muir-Torre syndrome. J. Am. Acad. Dermatol..

