# Supplementary material for: Sebaceous Carcinoma: A Retrospective Multicenter Analysis of 213 Cases
Source: Cancers (Basel). 2026 Apr 14;18(8):1245. doi: 10.3390/cancers18081245 (PMC13114816; doi:10.3390/cancers18081245)
Supplement: Supplementary file 1 [file cancers-18-01245-s001.zip › cancers-4237201_supplementary_figures.pdf]

Figure S1

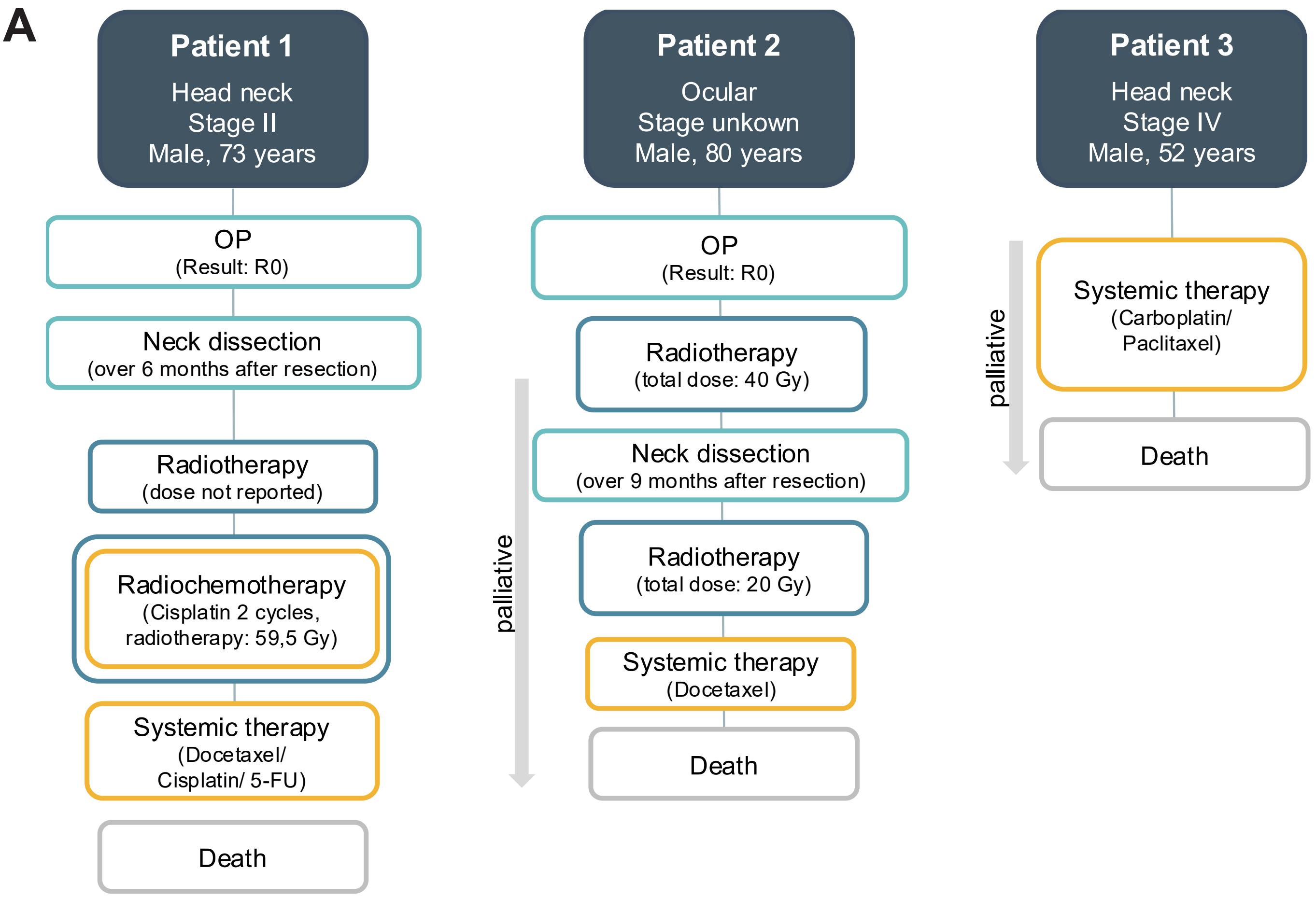

Figure S1: Flow chart of patients with systemic therapy

A. Flow chart showing the treatment schedule of patients that underwent systemic therapy in chronological sequence. Two patients received systemic therapy for SC with nodal involvement. One male patient suffered from metastasized SC with lung metastasis. Primary tumor thickness of the stage IV SC patient was >T2c. He died 12 months after the diagnosis was reported to the registry.

Figure S2

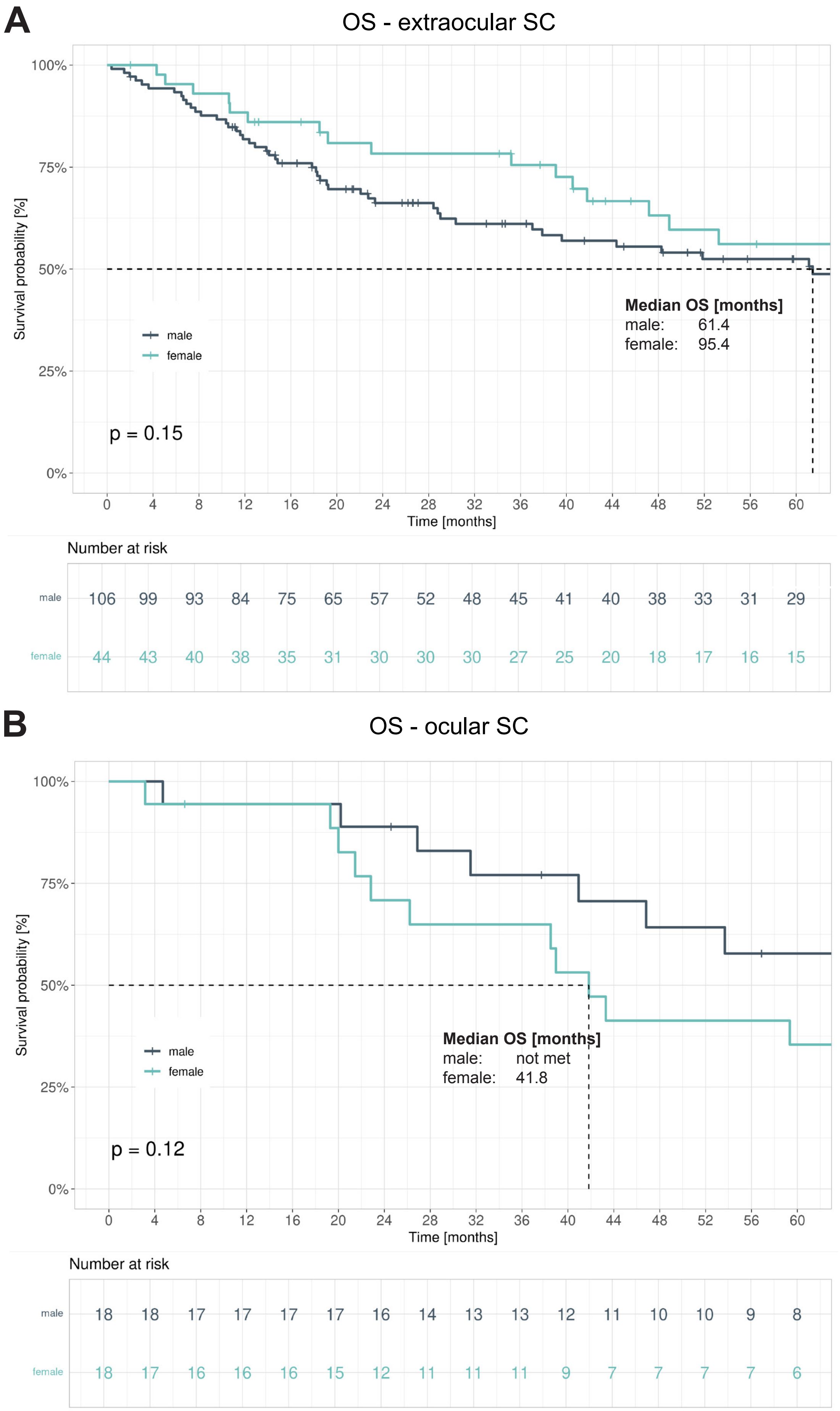

Figure S2: Gender-specific OS of extraocular and ocular SC patients
A. The 5-year OS of patients with extraocular SC was compared among males and females. Follow-up started from the date of diagnosis of SC. Kaplan-Meier curves are shown, male patients are presented in dark turquoise, and female patients in bright turquoise. The curves were compared by log-rank tests (*P* = 0.150).
B. The 5-year OS of patients with ocular SC was compared among males and females. Follow up started from the date of diagnosis of SC. Kaplan-Meier curves are shown, male patients are presented in dark turquoise, and female patients in bright turquoise. The curves were compared by log-rank tests (*P* = 0.120).

Figure S3

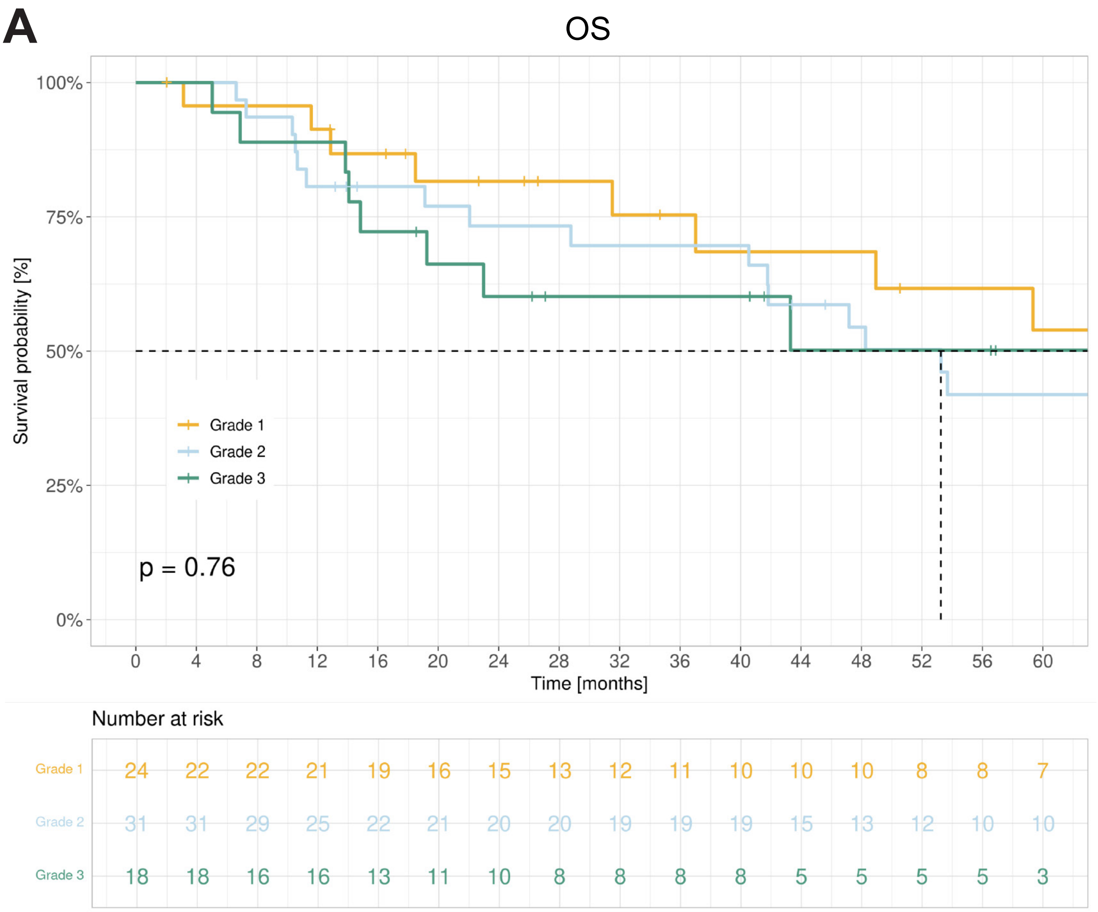

Figure S3: Sebaceous carcinoma. Influence of histologic grade on OS.
A. The 5-year OS of patients with SC grade one to three were analyzed. Follow-up started from the date of diagnosis of SC. Kaplan-Meier curves are shown, SC with grade one are presented in orange, grade two in bright blue and grade three in green. The curves were compared by log rank tests (*P* = 0.76).

Figure S4

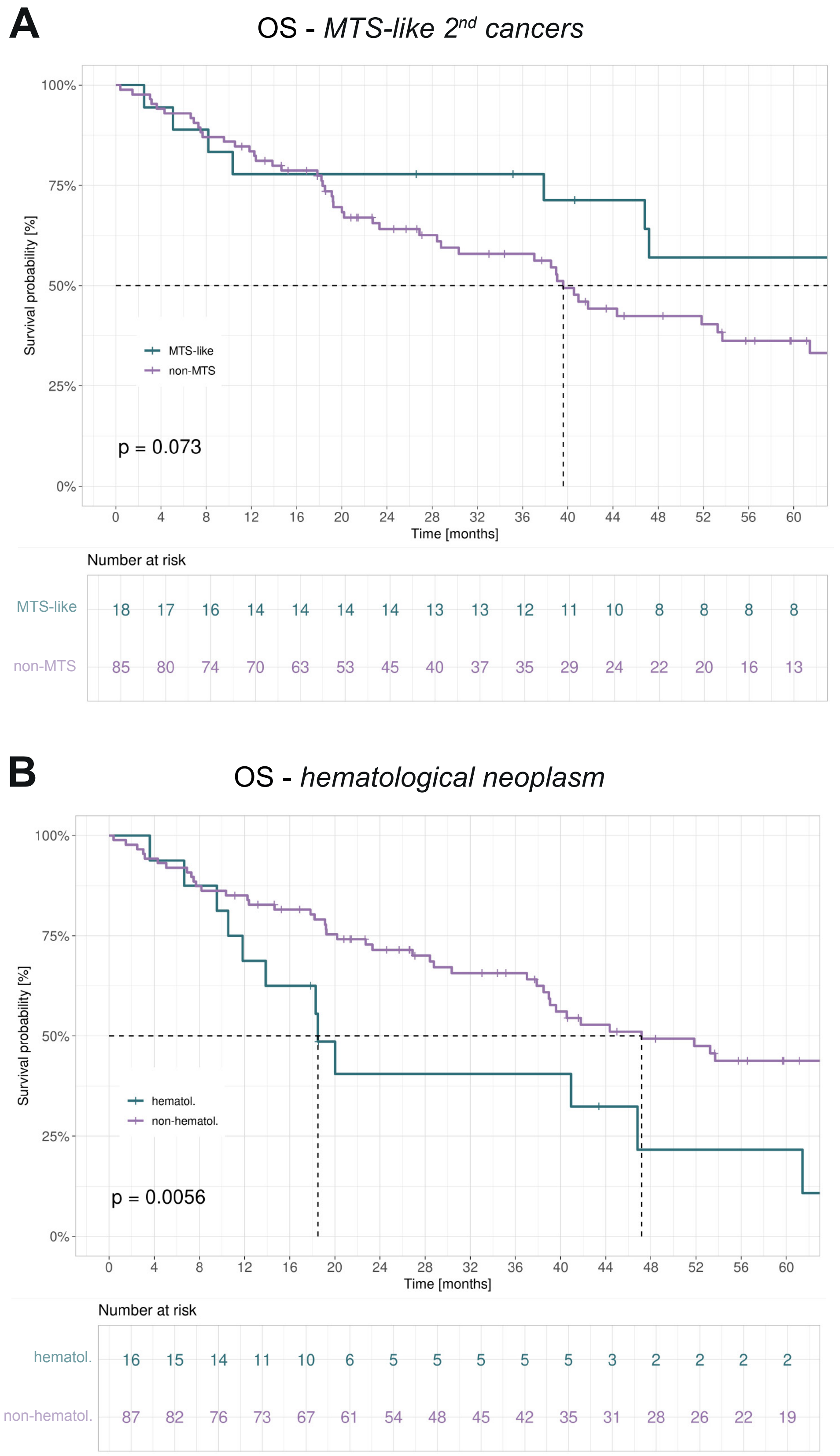

Figure S4: Prior cancer history and influence on disease course of SC
A. The influence of MTS-like cancers on 5-year OS of patients with SC has been investigated. Follow-up started from the date of diagnosis of SC. Kaplan-Meier curves are shown, SC with MTS-like cancers in the history are presented in dark turquoise, and patients with different secondary malignancies (non-MTS) are presented in purple. Mean age of MTS-like patients: 70.1 years. Mean age of patients with other secondary malignancies: 78.9 years (**Suppl. Table 9**). The curves were compared by log rank tests (*P* = 0.073). **B.** The influence of hematological malignan-cies on 5-year OS of patients with SC has been investigated. Follow-up started from the date of diagnosis of SC. Kaplan-Meier curves are shown, SCs with a history of hematological malignancies (hematol.) are presented in dark turquoise, and patients with a history of other cancers (non-hematol.) are presented in purple. Mean age of patients with hematological malignancies: 81.6 years. Mean age of patients with other secondary malignancies: 76.5 years (**Suppl. Table 10**). The curves were compared by log rank tests (*P* = 0.0056).
