# Supplementary material for: Sebaceous Carcinoma: A Retrospective Multicenter Analysis of 213 Cases
Source: Cancers (Basel). 2026 Apr 14;18(8):1245. doi: 10.3390/cancers18081245 (PMC13114816; doi:10.3390/cancers18081245)
Supplement: Supplementary file 1 [file cancers-18-01245-s001.zip › cancers-4237201_supplementary_tables.pdf]

Supplementary Tables

Suppl. Table S1: Grouping according to ICD-10 codes

ICD-Code	Localization
C44.0	Skin of the lips (excluding the lip and vermilion border C00.-)
C44.1	Skin of the eyelid, including the canthus
C44.2	Skin of the ear and external auditory canal
C44.3	Skin of other and unspecified parts of the face
C44.4	Scalp and skin of the neck
C44.5*	Skin of the trunk
C44.6	Skin of the upper limb, including the shoulder
C44.7	Skin of the lower limb, including the hip
C44.8	Skin, involving multiple overlapping areas
C44.9	Malignant neoplasm of the skin, unspecified
C51.9	Vulva, unspecified
C63.2	Malignant neoplasm of the scrotum

Suppl. Table S2: Cases with SC in stage III

ICD-10 code	Localization	Gender	Age	Therapy	Death (Time from date of diagnosis to death)
C44.3	nose to cheek	Male	89	Op, Rx	Yes (< 29 months)
C44.6	Upper limb/ shoulder	Male	88	Op	Yes (< 11 months)
C44.7	Lower limb/ Hip	Male	89	Op	Yes (< 20 months)
C44.3	Temple	Male	63	Op	-
C44.3	Nose	Female	71	Op	-

Op = Operation; Rx = Radiotherapy

Suppl. Table S3: Cases with SC in stage IV

ICD-10 code	Localization	Gender	Age	Therapy	Death (Time from date of diagnosis to death)
C44.4	Scalp/ Neck	Male	52	Sys	Yes (< 12 months)
C44.3	Infraorbital	Female	67	Rx	-

Rx = Radiotherapy; Sys = systemic therapy

Suppl. Table S4: Cases with LN excision

Procedure	Number of patients (%)	Localization		Stage
		Extracular	Ocular	
SLN biopsy	5 (2%)	5	-	I (n = 3)<br>No data (n = 2)
LN dissection	4 (2%)	2	2<br>(T ≥ 2)	III (n = 2)<br>No data (n = 2)
Not reported	204 (96%)			

Suppl. Table S5: Cases with radiotherapy

Procedure	Number of patients (%)	Localization		Stage	R status	Other
		Periocular	Ocular			
Definitive radiotherapy	2 (1 %)	2	-	III (n = 1)<br>IV (n = 1)		
Adjuvant radiotherapy	4 (2 %)	3	1	I (n = 1)<br>II (n = 1)<br>unknown (n = 2)	R0<br>(n = 4)	perineural invasion (n = 2)
Not reported	207 (97 %)					

Suppl. Table S6: Cases with multiple SC

Case	Localization	Time between diagnoses	Therapy	Stage	Gender	Age (at first SC diagn.) [years]	Previous cancers
1	Ear	All at first presentation	OP + R0	I	Male	74	C44 (NMSC)
	Upper lip		OP + R1	I			
	Scalp		OP + R1	I			
2	Preauricular	All at first presentation	OP + R0	I	Male	72	-
	Scalp		OP + R0	I			
3	Chest		OP + R0	I	Male	99	

	Scalp	All at first presentation	OP + R0	I			C18 (Colon), C44 (NMSC), C49 (other connective or soft tissue)
4	Neck	All at first presentation	OP + R0	I	Male	77	C44 (NMSC)
	Lower thigh		OP + R0	I			
5	Back	All at first presentation	-	I	Male	56	C18 (Colon)
	Lower thigh		-	I			
6	Jaw	All at first presentation	OP + R1	I	Male	77	C44 (NMSC), C91 (Lymphatic leukemia)
	Retroauricular		OP + R1	I			
	Frontal		OP + R0	I			
7	Neck	17 Months	OP + R0	-	Male	86	C44 (NMSC)
	Upper lip		OP + R0	-			
8	Scapula	26 or 28 Months	OP + R0	I	Male	74	C44 (NMSC)
	Chest		OP + R0	I			
	Lower thigh		OP + R0	I			
9	Cheek	All at first presentation	OP + R0	I	Female	90	-
	Upper lip		OP + R0	I			
	Nose/ infraorbital		OP + R0	I			

10	Ankle	91 Months	OP + R0	I	Female	76	C44 (NMSC)
	Neck		OP + R0	I			

Suppl. Table S7: Cases with SC and at least another malignancy, apart from skin tumors

**Cancer**	**Cases**
Tonsil	1
Colon + Breast	1
Colon + Prostate	1
Colon + Lymphatic leukemia	1
Rectal	1
Anal + Urogenital tract	1
Bile duct + pancreatic	1
Pancreatic	1
Lung	1
Lung + bladder	1
Lung + Hodgkin lymphoma	1
Bladder	1
Bladder + prostate	1
Thyroid gland	1

Cancer of unkown primary	1
Hodgkin lymphoma	1
Non-follicular lymphoma<br><br>+ lymphatic leukemia	1
Immunoproliferative disease (C88)	1
Multiple myeloma	1
Gastric + Colon	2
Colon	2
Other, not-specified	2
Breast	3
Prostate	6

MTS-like: colon, rectal, anal, bileduct, bladder, stomach
Hematological neoplasms: lymphatic leukemia, Hodgkin lymphoma, non-follicular lymphoma, immunoproliferative diseases, multiple myeloma

Suppl. Table S8: Cases with SC and at least another malignancy, including skin tumors (C44)

Cancer	Cases
Duodenal	1
Pancreatic	1
Laryngeal	1
Prostate	1
Eye	1
Uterus	1
Ovarial	1
Penis	1
Melanoma + prostate + lymphoma	1
Kidney	1
Ureter	1
Cancer of unkown primary	1
T cell lymphoma	1
Melanoma + Non-Hodgkin lymphoma	1
immunoproliferative disease (C88)	1
Lymphatic leukemia + prostate	1
Lymphatic leukemia + lymphoma + immunoproliferative disease (C88)	1

Myeloid leukemia	1
Lung	2
Lymphatic leukemia	2
Colon	3
Non-follicular lymphoma	4

MTS-like: duodenum, ovary, kidney, ureter

Hematological neoplasms: lymphoma, T-cell lymphoma, Non-Hodgkin lymphoma, immunoproliferative diseases, lymphatic leukemia, myeloid leukemia, non-follicular lymphoma

Suppl. Table S9: Baseline characteristics of MTS-like and non-MTS cohorts

**Variable**		**Overall**	**MTS-like**	**non-MTS**	***p* value**
n		103	18	85	
Age (mean)		77.32	70.11	78.85	**0.002**
Age (median)		80	70.5	81	
Age group	45-49	1 (1.0 %)	0 (0.0 %)	1 (1.2 %)	**0.019**
	50-54	3 (2.9 %)	2 (11.1 %)	1 (1.2 %)	
	55-59	4 (3.9 %)	2 (11.1 %)	2 (2.4 %)	
	60-64	9 (8.7 %)	2 (11.1 %)	7 (8.2 %)	

	65-69	6 (5.8 %)	3 (16.7 %)	3 (3.5 %)	
	70-74	10 (9.7 %)	3 (16.7 %)	7 (8.2 %)	
	75-79	12 (11.7 %)	2 (11.1 %)	10 (11.8 %)	
	80-84	33 (32.0 %)	2 (11.1 %)	31 (36.5 %)	
	> 84	25 (24.3 %)	2 (11.1 %)	23 (27.1 %)	
Gender	m	77 (74.8 %)	12 (66.7 %)	65 (76.5 %)	0.568
	f	26 (25.2 %)	6 (33.3 %)	20 (23.5 %)	
Diagnosis (ICD-10)	C44.0	1 (1.0 %)	0 (0.0 %)	1 (1.2 %)	0.157
	C44.1	13 (12.6 %)	2 (11.1 %)	11 (12.9 %)	
	C44.2	8 (7.8 %)	1 (5.6 %)	7 (8.2 %)	
	C44.3	38 (36.9 %)	5 (27.8 %)	33 (38.8 %)	
	C44.4	18 (17.5 %)	2 (11.1 %)	16 (18.8 %)	
	C44.5	14 (13.6 %)	6 (33.3 %)	8 (9.4 %)	
	C44.59	1 (1.0 %)	0 (0.0 %)	1 (1.2 %)	
	C44.6	4 (3.9 %)	0 (0.0 %)	4 (4.7 %)	
	C44.7	2 (1.9 %)	0 (0.0 %)	2 (2.4 %)	
	C44.9	3 (2.9 %)	2 (11.1 %)	1 (1.2 %)	
	C63.2	1 (1.0 %)	0 (0.0 %)	1 (1.2 %)	

Grading	1	10 (29.4 %)	1 (16.7 %)	9 (32.1 %)	0.700
	2	16 (47.1 %)	3 (50.0 %)	13 (46.4 %)	
	3	8 (23.5 %)	2 (33.3 %)	6 (21.4 %)	
Tumor status	T1	61 (83.6 %)	9 (90.0 %)	52 (82.5 %)	0.704
	T2	8 (11.0 %)	1 (10.0 %)	7 (11.1 %)	
	T3	4 (5.5 %)	0 (0.0 %)	4 (6.3 %)	
Nodal status	N0	61 (96.8 %)	9 (100.0 %)	52 (96.3 %)	1.000
	N1	2 (3.2 %)	0 (0.0 %)	2 (3.7 %)	
Metastases	M0	60 (100.0 %)	9 (100.0 %)	51 (100.0 %)	
Stage (UICC)	I	47 (82.5 %)	8 (88.9 %)	39 (81.2 %)	0.589
	II	5 (8.8 %)	1 (11.1 %)	4 (8.3 %)	
	III	5 (8.8 %)	0 (0.0 %)	5 (10.4 %)	
Subgroup localization	Extra-ocular	90 (87.4 %)	16 (88.9 %)	74 (87.1 %)	1.000
	ocular	13 (12.6 %)	2 (11.1 %)	11 (12.9 %)	
Tumor thickness of primary SC (±SD)		4.08 (3.74)	5.70 (3.98)	3.43 (3.65)	0.325

Suppl. Table S10: Baseline characteristics of SC patients with a history of hematological and non-hematological neoplasms

Variable		Overall	Hematological	non-hemato-logical	*p* value
n		103	16	87	
Age (mean)		77.32	81.62	76.53	0.084
Age (median)		80	81	80	
Age group	45-49	1 (1.0 %)	0 (0.0 %)	1 (1.1 %)	0.516
	50-54	3 (2.9 %)	0 (0.0 %)	3 (3.4 %)	
	55-59	4 (3.9 %)	0 (0.0 %)	4 (4.6 %)	
	60-64	9 (8.7 %)	0 (0.0 %)	9 (10.3 %)	
	65-69	6 (5.8 %)	0 (0.0 %)	6 (6.9 %)	
	70-74	10 (9.7 %)	1 (6.2 %)	9 (10.3 %)	
	75-79	12 (11.7 %)	3 (18.8 %)	9 (10.3 %)	
	80-84	33 (32.0 %)	8 (50.0 %)	25 (28.7 %)	
	> 84	25 (24.3 %)	4 (25.0 %)	21 (24.1 %)	
Gender	m	77 (74.8 %)	13 (81.2 %)	64 (73.6 %)	0.736
	f	26 (25.2 %)	3 (18.8 %)	23 (26.4 %)	
Diagnosis (ICD-10)	C44.0	1 (1.0 %)	0 (0.0 %)	1 (1.1 %)	0.402
	C44.1	13 (12.6 %)	3 (18.8 %)	10 (11.5 %)	

	C44.2	8 (7.8 %)	1 (6.2 %)	7 (8.0 %)	
	C44.3	38 (36.9 %)	7 (43.8 %)	31 (35.6 %)	
	C44.4	18 (17.5 %)	3 (18.8 %)	15 (17.2 %)	
	C44.5	14 (13.6 %)	0 (0.0 %)	14 (16.1 %)	
	C44.59	1 (1.0 %)	1 (6.2 %)	0 (0.0 %)	
	C44.6	4 (3.9 %)	1 (6.2 %)	3 (3.4 %)	
	C44.7	2 (1.9 %)	0 (0.0 %)	2 (2.3 %)	
	C44.9	3 (2.9 %)	0 (0.0 %)	3 (3.4 %)	
	C63.2	1 (1.0 %)	0 (0.0 %)	1 (1.1 %)	
Grading	1	10 (29.4 %)	2 (33.3 %)	8 (28.6 %)	0.906
	2	16 (47.1 %)	3 (50.0 %)	13 (46.4 %)	
	3	8 (23.5 %)	1 (16.7 %)	7 (25.0 %)	
Tumor status	T1	61 (83.6 %)	7 (63.6 %)	54 (87.1 %)	0.131
	T2	8 (11.0 %)	3 (27.3 %)	5 (8.1 %)	
	T3	4 (5.5 %)	1 (9.1 %)	3 (4.8 %)	
Nodal status	N0	61 (96.8 %)	7 (87.5 %)	54 (98.2 %)	0.595
	N1	2 (3.2 %)	1 (12.5 %)	1 (1.8 %)	
Metastases	M0	60 (100.0 %)	8 (100.0 %)	52 (100.0 %)	
Stage (UICC)	I	47 (82.5 %)	5 (62.5 %)	42 (85.7 %)	0.184
	II	5 (8.8 %)	1 (12.5 %)	4 (8.2 %)	

	III	5 (8.8 %)	2 (25.0 %)	3 (6.1 %)	
Subgroup localization	Extra-ocular	90 (87.4 %)	13 (81.2 %)	77 (88.5 %)	0.694
	ocular	13 (12.6 %)	3 (18.8 %)	10 (11.5 %)	
Tumor thickness of primary SC (±SD)		4.08 (3.74)	1.96 (0.63)	4.43 (3.94)	0.408
